# Efficacy and safety of Nefecon in IgA nephropathy: real world clinical practice

**DOI:** 10.3389/fimmu.2026.1761804

**Published:** 2026-02-05

**Authors:** Zihan Zhai, Zhibin Huang, Liuwei Wang, Lu Yu, Rong Gou, Yulin Wang, Qiuhong Li, Yanhong Guo, Lin Tang

**Affiliations:** Department of Nephropathy, The First Affiliated Hospital of Zhengzhou University, Zhengzhou, Henan, China

**Keywords:** CKD progression, EGFR, IgA nephropathy, Nefecon, proteinuria

## Abstract

**Introduction:**

The targeted-release budesonide formulation (Nefecon) addresses IgA nephropathy (IgAN) by inhibiting mucosal immune dysregulation in gut-associated lymphoid tissue (GALT), leading to reduced production of galactose-deficient IgA1 (Gd-IgA1). Randomized controlled trials (NEFIGAN, NefIgArd) have shown that Nefecon effectively decreases proteinuria and decelerates the progression of chronic kidney disease (CKD) in patients with IgA nephropathy (IgAN). However, evidence in real-world clinical settings and high-risk subgroups remains limited.

**Methods:**

We performed a retrospective cohort study involving 60 IgAN patients treated with Nefecon (16 mg/day) for no less than 6 months at the First Affiliated Hospital of Zhengzhou University between October 2024 and November 2025. The study focused on evaluating alterations in proteinuria and estimated glomerular filtration rate (eGFR). Subgroup analyses were further carried out according to the use of concomitant immunosuppressive therapy, baseline proteinuria levels, and baseline renal function.

**Results:**

Proteinuria decreased significantly after 4 and 6 months of Nefecon treatment (median reduction 31.9% and 43.5%, respectively; both p < 0.001), while eGFR remained stable. Patients receiving Nefecon plus glucocorticoid/immunosuppressive therapy achieved greater proteinuria reduction than those on Nefecon monotherapy (48.1% *vs*. 35.8% at 6 months, p=0.04). Notably, patients with a baseline eGFR < 35 mL/min/1.73 m² showed a 38.9% reduction in proteinuria (p=0.002) without additional renal deterioration. Nefecon was well tolerated, with adverse events being mild.

**Conclusion:**

In routine clinical practice, Nefecon effectively reduces proteinuria and preserves renal function in IgAN, even in patients excluded from randomized trials. The combination with immunosuppressive therapy may provide additive benefit that requires further validation. These findings extend trial results to real-world settings and highlight Nefecon as a practical treatment option for high-risk IgAN patients.

## Introduction

IgA nephropathy (IgAN) is the most common primary glomerulonephritis worldwide and a leading cause of end-stage kidney disease. In China (1), IgAN accounts for nearly half of biopsy-confirmed glomerular diseases, with up to 50% of patients progressing to kidney failure within 10–15 years (2, 3). Despite the high burden, current therapies such as glucocorticoids and immunosuppressants carry limited efficacy and substantial toxicity (1), leaving a major unmet need for safer, more effective interventions.

Recent studies emphasize mucosal immunity’s involvement in IgAN pathogenesis, especially the excessive production of galactose-deficient IgA1 in gut-associated lymphoid tissue (4–6). Nefecon, an oral targeted-release budesonide formulation, locally targets the distal ileum to directly suppress the pathogenic process. Phase 2b/3 trials (NEFIGAN, NefIgArd) showed that Nefecon effectively decreases proteinuria and maintains renal function (5–7), resulting in its endorsement in the 2025 KDIGO guideline for high-risk IgAN (8).

However, randomized trials often exclude patients with advanced CKD, recent or concurrent immunosuppressant use, or poor treatment adherence. Consequently, the real-world effectiveness and safety of Nefecon, particularly in Asian populations and high-risk subgroups, remain unclear. We performed a retrospective study on a Chinese cohort of IgAN patients, including those with eGFR <35 mL/min/1.73 m² undergoing immunosuppressive therapy, to assess the clinical utility of Nefecon in real-world settings.

## Materials and methods

### Study framework and subjects

**Figure 1:**
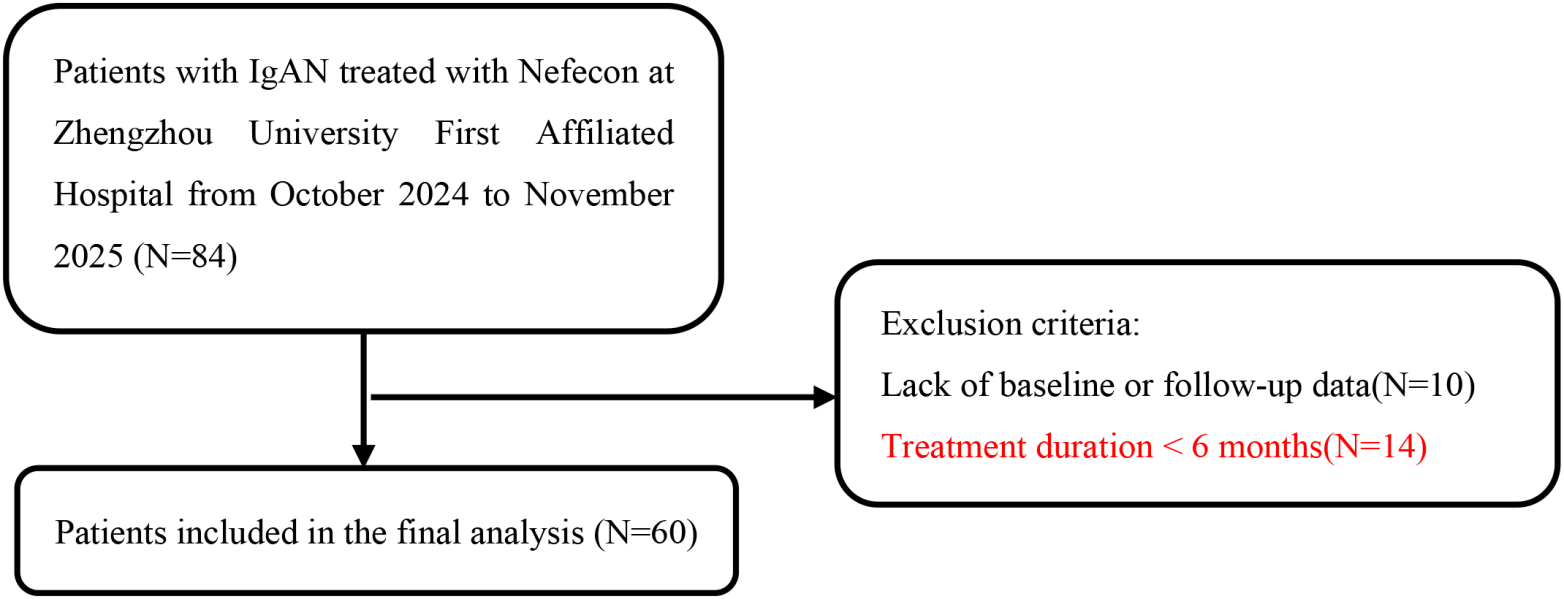
Study inclusion flowchart. IgAN, IgA nephropathy.

At the First Affiliated Hospital of Zhengzhou University, this retrospective study involved 84 patients with biopsy-confirmed IgA nephropathy who were treated with Nefecon between October 2024 and November 2025. The inclusion criteria included: (1) Primary IgAN verified through renal biopsy; (2) Age between 18 and 70 years; (3) The proteinuria was at least 0.5 g/d and eGFR of at least 25 mL/min/1.73m² at Nefecon treatment initiation; (4) Daily oral administration of 16 mg Nefecon. Exclusion criteria include: (1) absence of baseline or follow-up data; (2) treatment duration of less than 6 months. A total of 60 IgAN patients treated with Nefecon were included (**Figure 1**). The study adhered to the Declaration of Helsinki Guidelines and was approved by the medical ethics committee of the First Affiliated Hospital of Zhengzhou University (number. 2025-KY-0919).

### Data collection

Baseline characteristics encompassed age, gender, body mass index (BMI), presence of diabetes and hypertension, mean arterial pressure (MAP), proteinuria, albumin, eGFR, serum creatinine, and uric acid (UA). Proteinuria was measured using 24-hour proteinuria at all time points for all patients. eGFR was calculated using the CKD-EPI formula (9). Proteinuria and eGFR were recorded at each follow-up. Data on medications, such as glucocorticoids, immunosuppressants, renin-angiotensin-aldosterone system inhibitors (RAASi), and sodium-glucose cotransporter 2 (SGLT2) inhibitors, were documented.

Renal biopsy specimens were scored using the Oxford MEST-C classification (10, 11) (M: mesangial hypercellularity; E: endocapillary hypercellularity; S: segmental glomerulosclerosis; T: tubular atrophy/interstitial fibrosis; C: crescents).

### Outcomes and follow-up

Primary outcomes were changes in proteinuria and eGFR during follow-up. Secondary outcomes encompassed the cumulative frequency of patients achieving a 50% reduction in proteinuria, along with subgroup analyses based on varying treatment regimens, baseline proteinuria levels, and renal function. Baseline (time 0) is defined as the initiation of Nefecon treatment. All subsequent data were revised as of November 30, 2025.

Safety endpoints were the incidence of adverse events (AEs), including hypertension, peripheral edema, muscle spasms, acne, headache, respiratory infections, glucose intolerance, and abnormal liver function. Severe adverse events (SAEs) were defined as all-cause death and other life-threatening conditions requiring hospitalization.

### Statistical analysis

Categorical data were expressed as numbers and percentages. Continuous data with normal distribution were presented as mean ± standard deviation (SD), while non-normally distributed data were reported as median and interquartile range. Group differences were analyzed using the independent-samples t-test for data with normal distribution and the Wilcoxon-Mann-Whitney test for data without normal distribution. Temporal variations in variables were assessed using the paired-samples t-test for data with normal distribution and the Wilcoxon signed-rank test for data without normal distribution. For the subgroups with a small sample size (eGFR < 35 mL/min/1.73 m²), we employed exploratory analysis and noted that the statistical power of these analyses was relatively low and needed to be verified in a larger sample group. SPSS 27.0 software facilitated the statistical analysis. Statistical tests were conducted as two-tailed, with significance set at p < 0.05.

## Results

### Characteristics of study population

From October 2024 to November 2025, 60 IgAN patients treated with Nefecon were included. The time between IgAN diagnosis and the start of Nefecon treatment was 24 months, with a range of 1 to 60 months. Patients were monitored bi-monthly, with a median follow-up duration of 4 [2, 6] months. **Table 1** presents the baseline characteristics. The mean age was 37.52 years, 47% were male, and the mean BMI was 23.44 kg/m².Initial proteinuria measured 1.74 [0.92, 2.96] g/day, while the baseline eGFR was 61.04 [37.98, 96.24] mL/min/1.73 m².

**Table 1:** Baseline characteristics of patients with IgAN treated with Nefecon (n=60).

Baseline characteristics of patients with IgAN treated with Nefecon (n=60)	
Age, years	37.52 ± 12.93
Gender, male/female	28/32
BMI, kg/m^2^	23.44 [21.08, 25.39]
History of diabetes mellitus, n (%)	8 (12.90%)
History of hypertension, n (%)	29 (48.33%)
History of hepatitis infection (n, %)	0 (0.0%)
Systolic blood pressure, mmHg	129.44 ± 13.53
Diastolic blood pressure, mmHg	82.71 ± 10.86
Proteinuria, g/d	1.74 [0.92, 2.96]
Proteinuria category, n (%)	
≥2.0 g/d	26 (43.33)
<2.0 g/d	34 (56.67)
Hematuria, RBCs/μL	39 [8, 127]
having haematuria, n (%)	48 (80.0)
no haematuria, n (%)	12 (20.0)
Serum albumin, g/L	38.00 [35.40, 41.65]
Serum creatinine, μmol/L	119.0 [77.0, 167.5]
eGFR, mL/min per 1.73m²	61.04 [37.98, 96.24]
eGFR category, n (%)	
≥60 mL/min per 1.73m²	31 (51.67)
≥25 to <60 mL/min per 1.73m²	29 (48.33)
≥25 to <35 mL/min per 1.73m²	12 (20.00)
Serum uric acid, μmol/L	340.5 [283.2, 392.0]
Hemoglobin, g/L	127.73 ± 21.11
HbA1c, %	5.40 [5.20, 5.50]
Drug combination during Nefecon treatment, n (%)	
Glucocorticoid/immunosuppressor	24 (40.00%)
Glucocorticoid	9 (15.00%)
CSA/FK506	1 (1.67%)
MMF	20 (33.33%)
CTX	1 (1.67%)
RAASi	42 (70.00%)
SGLT2 inhibitors	10 (16.67%)
Oxford classification	
M0/M1	21/39
E0/E1	22/38
S0/S1	18/42
T0/T1/T2	36/14/10
C0/C1/C2	23/31/6

Data are presented as n (%), mean ± SD or median (IQR). eGFR was determined using the Chronic Kidney Disease.

Epidemiology formula. The Oxford classification was developed by the Working Group of the International IgA Nephropathy.

Network and the Renal Pathology Society.

IgAN, Immunoglobulin A nephropathy; BMI, body mass index; RBC, red blood cell; eGFR, estimated glomerular filtration rate; HbA1c, Hemoglobin A1c; CSA, cyclosporin A; FK506, tacrolimus; MMF, mycophenolate mofetil; CTX, cyclophosphamide; RAASi, renin-angiotensin-aldosterone system inhibitor; SGLT2 inhibitors, sodium-glucose cotransporter 2 inhibitors.

### Treatment regimens

All patients received treatment with Nefecon at a dose of 16mg. The expected duration of Nefecon treatment was 9 months. 24 (40.00%) patients were also given Glucocorticoid/immunosuppressor therapy, 42 (70.00%) patients were given RAASi therapy, and 10 (16.67%) patients were given SGLT2i therapy, with the dosage being the maximum or tolerated dose.

### Primary outcomes

**Figure 2:**
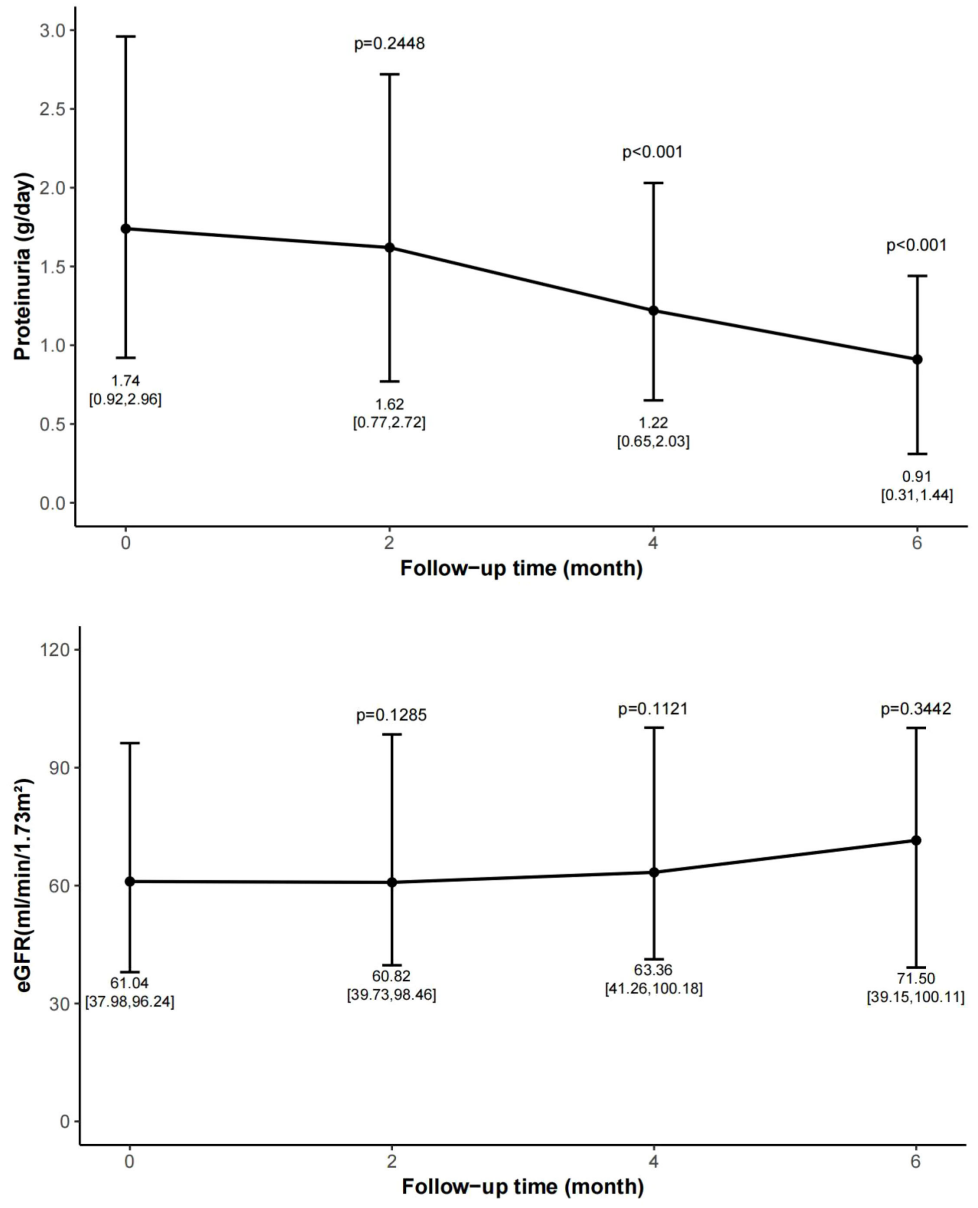
Change of proteinuria and eGFR during follow-up period. Data are expressed as median, upper quartile, and lower quartile. Follow-up was conducted every two months and compared with the baseline data. eGFR, estimated glomerular filtration rate.

The changes in proteinuria and eGFR over time are shown in **Figure 2**.

At month 2, the proteinuria decreased from 1.74 [0.92, 2.96] to 1.62 [0.77, 2.72] g/day, with a reduction rate of 16.6% [-37.7, 39.9] (p=0.24). At month 4, the proteinuria dropped to 1.22 [0.65, 2.03] g/day, representing a reduction rate of 31.9% [-15.5, 48.1] (p<0.001). At month 6, the proteinuria decreased to 0.91 [0.31, 1.44] g/day (p<0.001), with a reduction rate of 43.5% [15.5, 68.3]. A statistically significant reduction in proteinuria was observed after 4 months of treatment.

The eGFR remained stable throughout the follow-up period. The eGFR measured at 2, 4, and 6 months were 60.82 [39.73, 98.46] mL/min/1.73 m² (p=0.12 *vs*. baseline), 63.36 [41.26, 100.18] mL/min/1.73 m² (p=0.11 *vs*. baseline), and 71.50 [39.15, 100.11] mL/min/1.73 m² (p=0.34 *vs*. baseline), respectively.

### Secondary outcomes

In 24 patients (40.00%), the reduction in proteinuria exceeded 50%, and in 19 patients (31.67%), proteinuria decreased to less than 0.5 g/day.

### Subgroup analyses

According to whether the patients received glucocorticoid/immunosuppressor treatment during the treatment with Nefecon, the patients were further divided into the Nefecon group (Group N, n=36, 60.00%) and the Nefecon plus glucocorticoid/immunosuppressor group (Group N+IS, n=24, 40.00%). Baseline proteinuria (1.62 [0.90, 2.49] *vs*. 2.39 [1.66, 3.57] g/day, p=0.13) and eGFR (72.92 [36.75, 102.30] *vs*. 58.69 [36.60, 84.39] mL/min/1.73 m², p=0.17) levels showed no significant differences between the groups.

The changes in proteinuria and estimated glomerular filtration rate (eGFR) over time in the two groups are presented in **Tables 2** and **3**, respectively. After 4 months of follow-up, proteinuria levels decreased significantly in both groups. At month 4, proteinuria in Group N decreased to 1.16 [0.63, 1.53] g/day (p=0.004), whereas that in Group N+IS decreased to 1.34 [0.60, 2.87] g/day (p=0.01). After 6 months, the reduction rate of proteinuria in Group N+IS was more pronounced (48.1% [20.7, 76.7] *vs*. 35.8% [23.9, 57.6], p=0.04) (**Table 2**). In addition, eGFR levels remained stable over time in both groups (**Table 3**).

**Table 2:** Proteinuria (g/d) and proteinuria reduction rate (%) during treatment compared with baseline data.

Variables	Baseline	2 month	P value	4 months	P value	6 months	P value
Total patients	1.74 [0.92, 2.96]	1.62 [0.77, 2.72] 16.6% [-37.7, 39.9]	0.24	1.22 [0.65, 2.03] 31.9% [-15.5, 48.1]	<0.001	0.91 [0.31, 1.44] 43.5% [15.5, 68.3]	<0.001
N(n=36)	1.62 [0.90, 2.49]	1.23 [0.70, 1.84] 13.6% [-9.6, 43.6]	0.09	1.16 [0.63, 1.53] 26.2% [4.8, 51.2]	0.004	0.89 [0.41, 1.28] 35.8% [23.9, 57.6]	0.007
N+IS(n=24)	2.39 [1.66, 3.57]	1.83 [0.71, 4.35] 16.9% [-23.1,51.6]	0.29	1.34 [0.60, 2.87] 32.6% [-0.8, 51.0]	0.01	0.87 [0.31, 2.78] 48.1% [20.7, 76.7]	<0.001
P Value	0.13	0.86		0.53		0.04	
Baseline proteinuria ≥ 2.0 g/day(n=34)	3.13 [2.67,4.64]	2.65 [1.70, 4.41] 18.7% [0.7, 50.4]	0.02	2.04 [1.33, 3.45] 37.1% 23.6, 54.5]	<0.001	1.56 [0.87, 3.64] 47.7% [31.6, 70.4]	<0.001
Baseline proteinuria < 2.0 g/day(n=26)	0.99 [0.69,1.61]	1.09 [0.63, 1.72] -5.8% [-60.2, 32.4]	0.36	0.88 [0.57, 1.31] 17.9% [-35.0, 45.8]	0.28	0.71 [0.37, 1.19] 27.3% [-18.7, 64.8]	0.02
P Value	<0.001	0.002		<0.001		0.003	
Baseline eGFR ≥ 60 mL/min/1.73m^2^(n=31)	1.32 [0.71, 1.96]	1.16 [0.68, 1.91] 11.6% [-26.2, 43.6]	0.83	0.95 [0.51, 1.41] 31.5% [-13.7, 47.2]	0.15	0.60 [0.37, 1.45] 39.6% [-5.2, 68.9]	0.005
Baseline eGFR < 60 mL/min/1.73m^2^(n=29)	2.80 [1.32, 4.62]	2.30 [1.13, 4.35] 17.0% [-18.3, 32.3]	0.18	1.50 [0.84, 2.13] 32.6% [12.1, 51.0]	<0.001	1.24 [0.87, 1.84] 40.7% [21.6, 65.4]	0.01
P Value	0.11	0.29		0.07		0.23	
Baseline eGFR < 35 mL/min/1.73m^2^(n=12)	3.05 [1.74,4.64]	2.70 [1.69, 4.32] 13.5% [-32.0, 52.7]	0.42	1.74 [1.23, 2.61] 31.5% [8.3, 48.1]	0.01	1.41 [1.06, 2.29] 38.9% [21.4, 58.2]	0.002

N group: patients treated without glucocorticoid/immunosuppressor during Nefecon treatment; N+IS group: patients treated with glucocorticoid/immunosuppressor during Nefecon treatment; eGFR, estimated glomerular filtration rate. Data are presented as median (IQR). Proteinuria reduction rate=(Baseline data - Follow-up data)/Baseline data * 100. Follow-up was conducted every two months and compared with the baseline data.

**Table 3:** The change in eGFR during treatment compared with baseline data.

Variables	Baseline	2 month	P value	4 months	P value	6 months	P value
Total patients	61.04 [37.98, 96.24]	60.82 [39.73, 98.46]	0.12	63.36 [41.26, 100.18]	0.11	71.50 [39.15, 100.11]	0.34
N(n=36)	72.92 [36.75, 102.30]	78.32 [41.19,104.08]	0.50	74.51 [41.60,108.21]	0.22	80.25 [40.41,107.98]	0.27
N+IS(n=24)	58.69 [36.60, 84.39]	57.42 [33.15,65.04]	0.31	54.36 [31.93,70.76]	0.25	59.12 [32.87,72.85]	0.7
Baseline proteinuria ≥ 2.0 g/day(n=34)	41.34 [31.20,61.21]	40.70 [26.92,51.94]	0.38	47.60 [29.36,70.05]	0.54	46.87 [28.71,75.81]	0.77
Baseline proteinuria < 2.0 g/day(n=26)	77.29 [54.57,100.99]	79.82 [57.66,102.54]	0.17	81.51 [58.98,106.02]	0.18	80.25 [53.12,106.07]	0.4
Baseline eGFR ≥ 60 mL/min/1.73m^2^(n=31)	95.04 [77.18,114.80]	98.41 [79.07,116.87]	0.93	98.49 [80.68,115.08]	0.34	98.60 [79.33,114.48]	0.89
Baseline eGFR < 60 mL/min/1.73m^2^(n=29)	36.86 [29.86, 46.56]	40.12 [29.90,48.70]	0.30	42.71 [29.36,49.76]	0.31	39.67 [28.71,51.67]	0.35
Baseline eGFR < 35 mL/min/1.73m^2^(n=12)	28.73 [26.68, 31.50]	28.77 [26.57, 36.63]	0.38	30.06 [24.11, 34.42]	0.42	30.58 [24.75, 35.17]	0.62

N group: patients treated without glucocorticoid/immunosuppressor during Nefecon treatment; N+IS group: patients treated with glucocorticoid/immunosuppressor during Nefecon treatment; eGFR, estimated glomerular filtration rate. Data are presented as median (IQR). eGFR was determined using the Chronic Kidney Disease Epidemiology formula. Follow-up was conducted every two months and compared with the baseline data.

Subgroup analyses were conducted based on baseline proteinuria levels (<2.0 *vs*. ≥2.0 g/d) and eGFR categories (≥60 *vs*. <60 mL/min/1.73 m²). The group with baseline proteinuria ≥2.0 g/d showed a significantly greater reduction in proteinuria (47.7% [31.6, 70.4] *vs*. 27.3% [-18.7, 64.8], p=0.003), whereas no significant difference was observed in proteinuria reduction between groups with varying baseline eGFR levels (39.6% [-5.2, 68.9] *vs*. 40.7% [21.6, 65.4], p=0.23) (**Table 2**).

Among 12 patients with a baseline eGFR < 35 mL/min/1.73 m², proteinuria decreased by 38.9% [21.4, 58.2] (p=0.002) during treatment (**Table 2**), while eGFR levels remained stable (**Table 3**). For this subgroup, we employed exploratory analysis and indicated that the statistical power of these analyses was relatively low and required validation in a larger sample group.

### Adverse events related to Nefecon

Most patients tolerated Nefecon well. The blood sugar control was stable (the HbA1c at baseline was 5.40%, and at 6 months it was 5.53%, p = 0.32), and no new diabetes cases occurred; the blood pressure was well controlled (systolic/diastolic blood pressure: at baseline it was 129.44/82.71 mmHg, and at 6 months it was 127.36/80.58 mmHg, p > 0.05); and there were no severe infections (only 4 cases of upper respiratory tract infections, accounting for 6.7%). No serious adverse events (AEs) were reported. Mild AEs included acne (13.3%), weight gain (11.7%), facial swelling (10.0%), fatigue (8.3%), limb pain (6.7%), hypertension (6.7%), menstrual disorders (6.7%), elevated blood sugar (5.0%), peripheral edema (5.0%), and hirsutism (3.3%). No one has stopped using Nefecon because of AEs.

## Discussion

The exact mechanisms underlying IgAN remain unclear. Emerging evidence suggests that the intestinal mucosal immune system and mucosal-derived Gd-IgA1 play a role in the pathogenesis of primary IgAN. In IgAN patients, elevated circulating Gd-IgA1 levels lead to autoantibody production and immune complex formation, which deposit in the glomerular mesangium. This activates the complement alternative or lectin pathways and inflammatory responses, resulting in kidney damage (4, 12). Nefecon acts on Peyer’s patches in the terminal ileum to decrease the production of Gd-IgA1 at its origin. The 2025 KDIGO Clinical Practice Guideline for the Management of Immunoglobulin A Nephropathy (IgAN) and Immunoglobulin A Vasculitis (IgAV) advises using Nefecon for IgAN patients at risk of disease progression (8). The NefIgArd Phase III trial demonstrated that Nefecon significantly reduces proteinuria in IgAN patients with eGFR>35 mL/min/1.73 m², while maintaining stable renal function and exhibiting no serious adverse reactions (6, 7).

Our research offers real-world evidence supporting the efficacy and tolerability of Nefecon in IgAN patients, supplementing findings from controlled clinical trials. Consistent with the NEFIGAN and NefIgArd trials, a significant reduction in proteinuria was observed after 4 months of follow-up, with median reduction rates of 31.9% at 4 months and 43.5% at 6 months. During the treatment process, although there was no statistical difference, eGFR did show an improvement.

Furthermore, our study extends these findings to patient groups often excluded from RCTs. First, we demonstrated that concomitant use of Nefecon with glucocorticoids or immunosuppressants produced greater reductions in proteinuria compared to Nefecon alone. This suggests an exploratory finding through complementary mechanisms — while Nefecon targets mucosal IgA production, immunosuppressants act on systemic immune pathways. Secondly, we noted advantages in patients with severe renal impairment (eGFR <35 mL/min/1.73 m²), a group previously excluded from key trials. In these patients, Nefecon reduced proteinuria without accelerating renal decline, consistent with recent retrospective reports (13), and underscoring its potential value even in late-stage disease.

Finally, 18 patients did not receive RAASi during Nefecon treatment. Potential reasons include 8 patients having a baseline eGFR below 30 mL/min/1.73 m², 2 patients experiencing rapid renal function decline, and 8 patients showing intolerance to blood pressure.

The N+IS subgroup exhibited heterogeneity in immunosuppressive regimens, We recognize that these factors introduce residual confounding that cannot be fully eliminated in a retrospective study. Thus, the observed greater proteinuria reduction in the N+IS group should be interpreted with caution. We emphasize that this potential additive benefit is preliminary and hypothesis-generating, not definitive evidence of synergy. To validate these findings, future prospective trials with standardized immunosuppressive protocols and rigorous randomization to treatment arms are essential. Such studies will better control for confounding variables and clarify whether the observed additive effect is truly attributable to the combination therapy or confounded by baseline disease severity and regimen heterogeneity.

Several limitations should be considered when interpreting our findings. The study’s retrospective, single-center design and limited sample size restrict its generalizability. The absence of a control group and short follow-up duration preclude firm conclusions regarding long-term renal function preservation (stable eGFR during follow-up). Moreover, we did not assess biomarker changes such as serum Gd-IgA1, which could provide mechanistic insights. We explicitly emphasize that the study’s findings are exploratory and hypothesis-generating, rather than definitive evidence of causal relationships. A multi-center prospective controlled trials with a large sample size and extended follow-up is necessary to confirm Nefecon’s clinical efficacy.

## Conclusion

In summary, this study fills an important knowledge gap by evaluating Nefecon in real-world Chinese IgAN patients, including high-risk groups often excluded from trials. The findings underscore Nefecon’s safety and efficacy as a therapeutic option, with exploratory evidence of potential additive benefits when combined with immunosuppressants. These results are hypothesis-generating, suggesting the need for future multicenter studies with extended follow-up to validate its long-term advantages and establish optimal combination strategies.

## Data Availability

The raw data supporting the conclusions of this article will be made available by the authors, without undue reservation.
